# Corn silk extract as a prebiotic exerts antihypertensive effects via gut microbiota modulation in hypertensive rats

**DOI:** 10.1128/spectrum.01442-25

**Published:** 2026-01-22

**Authors:** Guixiang Yao, Tongxue Zhang, Zihan Qin, Yu Wang, Junfei Gu, Chuan He, Jiajia Jin

**Affiliations:** 1 State Key Laboratory for Innovation and Transformation of Luobing Theory, Key Laboratory of Cardiovascular Remodeling and Function Research of MOE, NHC, CAMS and Shandong Province, Department of Cardiology, Qilu Hospital of Shandong University 91623, Jinan, China; 2 Department of Geriatric Medicine, Qilu Hospital of Shandong University 91623, Jinan, Shandong, China; 3 Cheeloo College of Medicine, Shandong University 66555, Jinan, China; 4 Affiliated Hospital of Shandong University of Traditional Chinese Medicine, Jinan, China; 5 Department of Endocrinology, the Second Affiliated Hospital of Wannan Medical College, Wuhu, China; Cleveland Clinic Lerner Research Institute, Cleveland, Ohio, USA

**Keywords:** corn silk extract, prebiotic, gut microbiota remodeling, hypertension, systemic inflammation, host health

## Abstract

Corn silk extract (CSE), a traditional medicinal food rich in polysaccharides, flavonoids, and saponins, has been used as a natural antihypertensive agent, but its mechanism remains unclear. This study aimed to evaluate whether CSE can lower blood pressure through gut microbiota modulation. Spontaneously hypertensive rats received oral CSE for 4 weeks, followed by a 4-week drug-free observation. The treatment significantly reduced blood pressure, increased microbial diversity, decreased the *Firmicutes*/*Bacteroidetes* ratio, and enriched beneficial genera, such as *Akkermansia* and *Lactobacillus*. These changes were accompanied by reduced serum lipopolysaccharide and pro-inflammatory cytokines, elevated nitric oxide (NO) levels, and restored endothelial function. Permutational multivariate analysis of variance (PERMANOVA) and correlation analyses showed that microbiota and inflammatory markers were more strongly associated with blood pressure improvements than urinary indices. Structural equation modeling suggested a potential mechanistic pathway involving gut microbiota-inflammation-NO regulation. Importantly, fecal microbiota transplantation using post-treatment donor samples reproduced the antihypertensive and anti-inflammatory effects, confirming the microbiota’s critical mediating role. These findings provide the first experimental evidence that CSE functions as a prebiotic to improve gut microbial balance and vascular health, offering a promising natural strategy for microbiota-targeted blood pressure control.

**IMPORTANCE:**

This study identifies corn silk extract (CSE) as a novel plant-derived prebiotic with antihypertensive effects mediated through gut microbiota modulation. Using a spontaneously hypertensive rat model, we demonstrated that CSE reshapes gut microbial composition, enhances microbial diversity, and promotes beneficial genera while reducing systemic inflammation and restoring nitric oxide (NO)-mediated vascular function. Importantly, fecal microbiota transplantation confirmed the causal role of gut microbiota in mediating these effects. These findings highlight a gut microbiota-inflammation-NO axis as a key pathway through which CSE regulates blood pressure. As a safe, accessible, and food-compatible intervention, CSE represents a promising strategy for non-pharmacological blood pressure management and broadens the application scope of prebiotics in cardiovascular health.

## INTRODUCTION

Hypertension is one of the most common chronic non-communicable diseases worldwide, affecting more than 1.1 billion adults globally, with its prevalence continuing to rise (1). As the leading risk factor for major health conditions, such as cardiovascular and cerebrovascular diseases and renal impairment, hypertension remains a major contributor to global morbidity and mortality (2). Although current clinical management primarily relies on antihypertensive medications, such as angiotensin-converting enzyme inhibitors and calcium channel blockers, these agents can effectively reduce blood pressure in the short term, but long-term blood pressure control remains suboptimal (3). Large-scale epidemiological surveys have shown that in developing regions such as China, the hypertension control rate remains below 20%, and most patients require combination therapy involving multiple medications (4). The efficacy of pharmacological treatment is often limited by poor adherence, adverse effects, and long-term dependence. Therefore, there is an urgent need to explore novel antihypertensive strategies that are safer, better tolerated, and mechanistically well-defined.

Against this background, non-pharmacological interventions have attracted increasing attention, particularly gut microbiota-targeted strategies, which can simultaneously modulate metabolic, immune, and inflammatory systems and have become a rising focus in chronic metabolic disease research. Multiple clinical and animal studies have shown that gut microbiota dysbiosis may contribute to elevated blood pressure by inducing systemic inflammation, reducing nitric oxide (NO) synthesis, or impairing intestinal barrier function (2). Furthermore, experimental approaches such as 16S rRNA gene sequencing, antibiotic-induced microbiota depletion, and fecal microbiota transplantation (FMT) have demonstrated the causal role and regulatory potential of the gut microbiota in hypertension pathogenesis (5). In our recent work, we found that high salt intake in rats induced gut microbial disturbances that exacerbated hypertension progression, while probiotic supplementation partially reversed this trend. In another study on pregnancy-induced hypertension (preeclampsia), FMT experiments confirmed the causal contribution of microbial dysbiosis to both blood pressure elevation and placental dysfunction, further supporting the role of host-microbiota interactions in hypertensive disorders (6, 7). However, most current studies remain at an observational level, focusing primarily on whether gut microbiota is involved in hypertension and fail to provide deeper insights into how targeted microbial modulation can be used for therapeutic intervention. In the area of nutritional interventions, research has largely centered on probiotics or standardized dietary fibers, with limited strategies and insufficient mechanistic understanding (8). Given this, the development of targeted interventions toward specific taxa or functional groups has emerged as a key direction in hypertension management. Compared with non-specific approaches, nutritional compounds with higher physiological compatibility and controllability are increasingly viewed as promising candidates for clinical translation.

Prebiotics, as nutritional factors capable of modulating gut microbiota composition and metabolic homeostasis, have increasingly been applied in non-pharmacological interventions for hypertension in recent years (9). Animal studies and preliminary clinical trials have demonstrated that supplementation with typical prebiotics, such as fructooligosaccharides, inulin, and resistant starch can effectively reduce blood pressure, potentially through regulating gut microbial function and improving host metabolic and immune status (10, 11). Although these studies provide strong proof-of-concept evidence, existing research has primarily focused on a few standardized oligosaccharides, with limited clinical trial sample sizes, short intervention durations, and low translational potential (12). Moreover, substantial inter-individual variability may affect the efficacy of prebiotic interventions, limiting their broad applicability in hypertensive populations (13). To enhance the feasibility and generalizability of prebiotic strategies, the development of naturally derived prebiotics with greater structural diversity, safety, and long-term dietary compatibility has become a critical area of research. Soluble polysaccharides and polyphenolic compounds derived from edible and medicinal plants—especially those from traditional food-medicine homologous resources—have emerged as promising candidates for the next generation of prebiotics due to their multifunctionality and wide availability (14). These compounds may regulate gut microbiota and host metabolic-immune functions via multiple pathways, yet most current findings remain at the level of trend observations, lacking systematic mechanistic validation.

Corn silk, the elongated stigmas of female maize flowers, is typically harvested at the immature stage and consumed fresh, dried, or as an extract. As a traditional medicinal food with a long history of use and excellent safety profile, corn silk is rich in polysaccharides, polyphenols, and flavonoids that have been shown in other studies to possess typical prebiotic properties (15). Accumulating evidence suggests that corn silk exhibits therapeutic potential in managing chronic metabolic disorders characterized by hyperglycemia and hyperlipidemia. For instance, corn silk extract (CSE) ameliorated streptozotocin-induced type one diabetes by reducing FBG levels and pancreatic injury, which was mediated through modulation of gut microbiota composition including *Firmicutes*, *Actinobacteria*, *Desulfobacterota*, and *Proteobacteria* and subsequent short-chain fatty acid (SCFA)-dependent regulation of inflammatory cytokines via the G-protein-coupled receptor 41 signaling pathway (16). Randomized controlled trials have demonstrated that corn silk decoction exhibits significant lipid-modulating effects in subjects with angina pectoris, characterized by an elevation in high-density lipoprotein cholesterol levels and concurrent reductions in serum total cholesterol, triglycerides, and low-density lipoprotein cholesterol concentrations (17). Additionally, preclinical studies have shown that corn silk polysaccharides can reduce not only blood glucose but also serum lipid levels, including total cholesterol and triglycerides, in diabetic rats (18). Corn silk has also been reported to exert antihypertensive, anti-inflammatory, and diuretic effects (19). However, current research on the antihypertensive effects of corn silk is extremely limited. Only a few studies have reported that CSE can lower systolic blood pressure (SBP) in hypertensive rats, primarily through the inhibition of angiotensin-converting enzyme activity (20). Notably, no studies have yet systematically evaluated the prebiotic potential of CSE or its mechanism of action in hypertension. Therefore, exploring its microbiota-modulating capacity is of great significance for advancing the development and application of novel plant-derived prebiotics in hypertension management.

Based on the above background and theoretical framework, this study aimed to systematically evaluate the antihypertensive effects of CSE in a spontaneously hypertensive rat (SHR) model and to determine whether these effects are mediated through modulation of gut microbiota composition and function. We further investigated whether its bioactive components possess prebiotic properties capable of restoring gut homeostasis, alleviating systemic inflammation, and enhancing NO signaling, ultimately contributing to blood pressure improvement. To elucidate these mechanisms, we employed 16S rRNA sequencing, serum inflammatory and NO marker assays, and FMT to validate the functional link between microbial alterations and host vascular outcomes. The results demonstrated that CSE lowered blood pressure by reshaping the gut microbiota and alleviating systemic inflammation, supporting its potential as a plant-derived prebiotic. This study provides new evidence on the mechanistic actions of corn silk and may expand its application in microbiota-targeted strategies for hypertension management.

## MATERIALS AND METHODS

### Animals and ethical approval

Male SHR were used as the animal model in this study. All animals were obtained from Beijing Vital River Laboratory Animal Technology Co., Ltd. (license number: SCXK [Jing] 2016-0006). At the beginning of the experiment, rats were 8 weeks old and weighed 220 ± 10 g. All animals were housed under specific pathogen-free conditions with a 12-h light/dark cycle at a controlled temperature of 22°C ± 2°C and relative humidity of 50% ± 10%, and were provided with free access to water and a standard chow diet.

### Preparation and component analysis of CSE and animal treatment

Fresh corn silk (*Zea mays* L.) was collected during the pollination stage, approximately 1–2 weeks after pollination, from conventional farmland in Licheng District, Jinan, China. According to the supplier’s standard production procedure, the material was washed, low-temperature dried (≈45°C), pulverized, and defatted using a food-grade organic solvent (n-hexane). The subsequent extraction was performed by the supplier using an aqueous-ethanol method commonly applied for obtaining polysaccharides, polyphenols, and flavonoids from corn silk. The final extract was concentrated under reduced pressure, spray-dried, and stored at −20°C until use. Major components were quantified as follows: total dietary fiber and soluble dietary fiber (enzymatic–gravimetric method, AOAC 991.43), total polyphenols (Folin–Ciocalteu method, expressed as gallic acid equivalents), total flavonoids (aluminum colorimetric method), and total saponins (vanillin–sulfuric acid method). The results of component analysis are summarized in Table 1.

**TABLE 1:** Major bioactive components of CSE (dry-weight basis)

Component	Content (g/100 g）
Total dietary fiber	51.2
Soluble dietary fiber	4.3
Saponins	6.9
Total polyphenols	3.6
Flavonoids	1.5

After a 1-month acclimation period, animals were randomly divided into two groups (*n* = 10 per group): a control group (SHR) and a CSE intervention group (CSE group). Rats in the CSE group received 300 mg/kg of CSE by daily oral gavage for four consecutive weeks, followed by a 4-week drug-free observation period to evaluate the persistence of antihypertensive effects. Rats in the control group received an equal volume of sterile saline during the corresponding treatment period. Fecal samples were collected from both groups prior to intervention and at the experimental endpoint (week 8) and were immediately snap-frozen and stored at −80°C until further gut microbiota analysis.

### Gut microbiota analysis based on 16S rRNA gene sequencing

Fecal samples were stored at −80°C until further processing. Microbial genomic DNA was extracted using the QIAamp Fast DNA Stool Mini Kit (Qiagen, Germany). The V3–V4 hypervariable region of the bacterial 16S rRNA gene was amplified using specific primers 338F (ACTCCTACGGGAGGCAGCAG) and 806R (GGACTACHVGGGTWTCTAAT). PCR amplicons were purified with AMPure XP magnetic beads (Beckman Coulter, USA) and subjected to high-throughput sequencing using the Illumina NovaSeq 6000 platform (PE250 mode). Raw sequencing data were quality-filtered using fastp (v0.23.2) and merged with FLASH (v1.2.11). Amplicon sequence variants (ASVs) were generated via the DADA2 pipeline within QIIME2. Taxonomic annotation of ASVs was performed using the built-in QIIME2 classifier against the SILVA 16S rRNA gene database (v138). Downstream analyses included alpha diversity (Shannon index), community structure (principal coordinate analysis, PCoA), genus-level abundance profiling (e.g., *Akkermansia*, *Lactobacillus*, *Escherichia-Shigella*), and calculation of the *Firmicutes*/*Bacteroidetes* (F/B) ratio. Functional prediction of microbial communities was conducted using PICRUSt2.

### Twenty four-hour urine collection

Twenty four-hour urine samples were collected using individual metabolic cages. Two days before the experimental endpoint (8 weeks), rats were transferred from their home cages to metabolic cages for a 24-h habituation period, during which no urine was collected and animals had free access to water and standard chow. Urine was then collected over the subsequent 24 h in the same cages. The metabolic cages were equipped with built-in meshes to separate urine from feces and prevent contamination. Blood pressure measurements were conducted on separate days in the home cages and were not performed during metabolic cage housing, thereby minimizing stress-related interference with hemodynamic outcomes. At the end of the collection period, urine samples were collected into tubes preloaded with mineral oil to prevent evaporation, and total urine volume was recorded immediately.

### Intestinal permeability assessment and terminal tissue collection

At the experimental endpoint (week 8), intestinal permeability was evaluated using fluorescein isothiocyanate–dextran (FITC–dextran, 4 kDa). Rats were fasted for 4 h with free access to water and then administered FITC–dextran dissolved in sterile PBS by oral gavage at a dose of 600 mg/kg body weight. Four hours after gavage, rats were deeply anesthetized with sodium pentobarbital (50 mg/kg, intraperitoneally, prepared as a 1% solution). Adequate depth of anesthesia was confirmed by the absence of pedal withdrawal and corneal reflexes. Animals were then euthanized by exsanguination via cardiac puncture, and blood samples were collected for serum preparation. Serum was separated by centrifugation at 3,000 × *g* for 10 min at 4°C, and FITC–dextran fluorescence was measured using a microplate reader (excitation 485 nm, emission 528 nm). FITC–dextran concentrations were calculated from a standard curve generated using serial dilutions prepared in blank rat serum. The same serum samples were used for LPS, ROS, and other cytokine measurements.

Immediately after blood collection, tissues, including the thoracic aorta and colon, were rapidly harvested from the same animals. Colonic tissues and thoracic aortas were gently rinsed with ice-cold PBS. Colonic tissues were fixed in paraformaldehyde for subsequent immunohistochemical analyses. A portion of the thoracic aorta was snap-frozen and stored at −80°C for subsequent molecular analyses, while the remaining thoracic aortic segments were immediately used for vascular reactivity and vasodilation experiments.

### FMT experimental design

Fresh fecal samples were collected at the experimental endpoint (week 8) from donor rats in the original SHR control group or the CSE-treated group of the primary animal experiment and were resuspended in sterile phosphate-buffered saline (PBS, pH 7.4), followed by centrifugation at 800 × *g* for 2 min at 4°C to remove large particulate matter. The supernatant was collected, and the centrifugation step was repeated 2–3 times to obtain a clarified bacterial suspension. To estimate the bacterial concentration, 1 μL of the suspension was serially diluted with PBS to 10⁻³, 10⁻⁶, and 10⁻⁹, and 10 μL from each dilution was plated onto LB agar plates. After incubation at 37°C for 24 h, colony-forming units (CFU) were counted and used to calculate the bacterial concentration in the original suspension. The final bacterial suspension was adjusted to 1×10⁹ CFU/mL in sterile PBS and used for subsequent FMT. Prior to FMT, intestinal microbiota depletion was performed by oral gavage of a broad-spectrum antibiotic cocktail once daily for 14 days. The cocktail contained vancomycin (100 mg/kg), neomycin sulfate (200 mg/kg, Solarbio #N8090-50g), metronidazole (200 mg/kg, Abcam #ab141218), and ampicillin (200 mg/kg, Solarbio #A8180-25g). After antibiotic treatment, rats were administered 1 mL of fecal microbiota suspension from the designated donor group via oral gavage every other day for 4 weeks. Rats were then further monitored for an additional 1 week before sacrifice. At the experimental endpoint (week 7), serum samples were collected, and thoracic aorta and colon tissues were harvested for subsequent molecular, biochemical, histological, and functional analyses. All procedures were performed under sterile conditions.

### Measurement of blood pressure and basic metabolic parameters

SBP was measured weekly using the tail-cuff method (BP-98A, Chengdu Taimeng Technology). Metabolic cages were used to monitor body weight, food and water intake. Twenty-four-hour urine samples were collected to determine urine volume and electrolyte concentrations (Na^+^, K^+^). In addition, serum creatinine, blood urea nitrogen (BUN), alanine aminotransferase (ALT), and aspartate aminotransferase (AST) were measured using an automated biochemical analyzer (Roche Cobas c311).

### Vascular functional assessment

Thoracic aortas were carefully isolated immediately after euthanasia. The entire thoracic segment (from the aortic arch to the diaphragm) was excised and placed in cold oxygenated Krebs–Henseleit solution. After removing surrounding fat and connective tissues under a stereomicroscope, the aorta was cut into 2–3-mm-long rings. Endothelium was preserved for acetylcholine-induced relaxation studies, which was confirmed by >70% relaxation in response to 10⁻⁵ mol/L acetylcholine after pre-contraction.

Each ring was mounted on a wire myograph (or organ bath system) under a resting tension of 1.5 g and equilibrated for 60 min at 37°C in Krebs solution continuously aerated with 95% O_₂_ and 5% CO_₂_. Rings were pre-contracted with phenylephrine (10⁻⁶ mol/L), and cumulative concentration–response curves were generated using acetylcholine (10⁻¹⁰–10⁻⁵ mol/L) to assess endothelium-dependent relaxation, and sodium nitroprusside (10⁻¹⁰–10⁻⁵ mol/L) to assess endothelium-independent relaxation (21).

### Assessment of inflammation and vascular function markers

Lipopolysaccharide (LPS), interleukin-6 (IL-6), tumor necrosis factor-α (TNF-α), and C-reactive protein (CRP) levels were measured using commercial ELISA kits. Nitric oxide (NO) in serum and thoracic aorta levels were determined using the Griess reagent method. The expression levels of endothelial nitric oxide synthase (eNOS) and its phosphorylated form (p-eNOS) of thoracic aortas were evaluated by Western blotting. β-actin was used as the internal control, and band intensities were quantified using ImageJ software. In addition, total RNA was extracted from thoracic aortic tissues, reverse-transcribed into cDNA, and Toll-like receptor 4 (TLR4) mRNA expression was quantified by real-time PCR using gene-specific primers (forward: 5′-AGCTTTGGTCAGTTGGCTCT-3′; reverse: 5′-CAGGAGCAGAGTTTCTCCAG-3′), with GAPDH as the reference gene.

### Immunohistochemical staining

Colonic tissues used for immunohistochemistry were collected from the same animals that were euthanized at the experimental endpoint for serum, vascular, and microbiota analyses. Rat colonic tissues were fixed in 4% neutral buffered paraformaldehyde for 24 h, paraffin-embedded, and sectioned (5 μm). After deparaffinization and antigen retrieval in citrate buffer (pH 6.0), sections were blocked with 5% goat serum and incubated overnight at 4°C with primary antibodies against Occludin (Rabbit monoclonal, 1:200, Abcam #ab216327) and ZO-1 (Mouse monoclonal, 1:150, Invitrogen #33-9100). HRP-conjugated secondary antibodies (1:500) and DAB visualization were performed, followed by hematoxylin counterstaining. Negative controls omitted primary antibodies. Protein expression was quantified using ImageJ by analyzing integrated optical density in three randomly selected fields per section.

### Statistical analysis

Data distribution was assessed using the Shapiro–Wilk test. Normally distributed data are expressed as mean ± SD and compared using Student’s *t*-test (or paired *t*-test) or one-way ANOVA followed by LSD post hoc test. When two independent factors and their interaction effects were simultaneously evaluated, two-way ANOVA followed by LSD post-hoc test was applied. Non-normally distributed data are presented as median (interquartile range, IQR) and analyzed using the Mann–Whitney U test or Kruskal–Wallis test followed by Dunn’s post hoc test. Linear regression was used to assess the contribution of multiple variables to blood pressure changes, with statistical significance set at *P* < 0.05. Permutational multivariate analysis of variance (PERMANOVA) was conducted using the adonis function in the vegan package in R, based on Bray–Curtis or Euclidean distance matrices, to evaluate the explanatory power of various factors on blood pressure variation. All statistical analyses and data visualization were performed using R version 4.3 (packages including vegan, lavaan, and ggplot2) and GraphPad Prism 9.

## RESULTS

### CSE effectively reduces blood pressure and alleviates systemic inflammation without affecting basic metabolism or organ function

**Fig 1:**
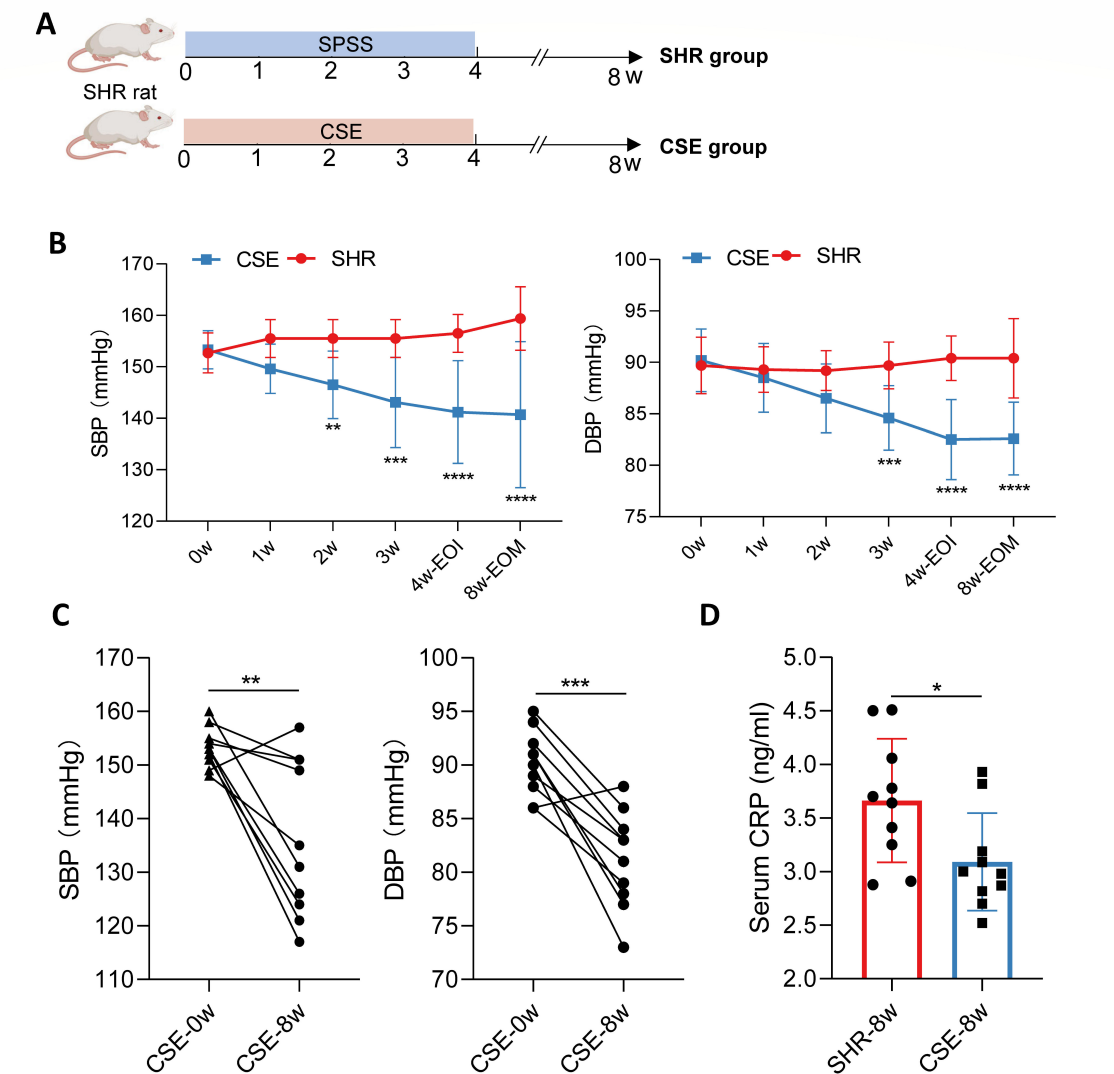
Experimental schematic and effects of CSE intervention on blood pressure and systemic inflammation in rats. (**A**) Schematic of the experimental protocol for CSE intervention in SHR rats. SPSS: Stroke-Physiological Saline Solution. (**B**) Changes in SBP (left) and DBP (right) over 8 weeks in the SHR and CSE groups. EOI: end of intervention; EOM: end of monitoring. (**C**) Comparison of SBP (left) and DBP (right) in the CSE group before (CSE-0w) and after 8 weeks of intervention (CSE-8w). Each line or pair of dots represents data from the same individual mouse. (**D**) Comparison of serum CRP levels between SHR (SHR-8w) and CSE (CS-8w) groups at week 8. SHR: spontaneously hypertensive rats; CSE: CSE-treated group. *n* = 10 per group. Data are presented as mean ± SD for panels **B and D**. Two-way ANOVA with LSD post-hoc test was used for panel **B**; paired Student’s *t*-test was used for panel **C**; two-tailed Wilcoxon rank-sum test was used for panel **D**. *: *P* < 0.05, **: *P* < 0.01, ***: *P* < 0.001, ****: *P* < 0.0001.

To evaluate the efficacy and long-term antihypertensive effects of CSE, this study administered CSE via gavage to SHR for 4 weeks, followed by a 4-week monitoring period after discontinuation to assess sustained blood pressure reduction (Fig. 1A). Results demonstrated that the CSE-treated group exhibited a continuous decrease in blood pressure throughout the 8-week experimental period, with significant differences compared to the SHR control group starting from week two and persisting until the endpoint (Fig. 1B). Self-controlled analysis further revealed that, compared to baseline, the CSE group showed significant reductions in SBP and diastolic blood pressure (DBP) at week 8 (Fig. 1C; *P* = 0.0056 and *P* = 0.0001, respectively), indicating a pronounced antihypertensive effect of CSE. As shown in Table 2, the CSE group exhibited significantly better renal function compared with the SHR control group, with no notable changes in liver function at week 8, indicating that CSE may protect against hypertension-induced renal impairment while demonstrating good biosafety. Notably, post-intervention serum CRP levels in the CSE group were significantly lower than those in the SHR control group (*P* < 0.05), and self-controlled analysis confirmed a significant reduction in CRP levels in the CSE group (*P* = 0.03) (Table 2; Fig. 1D), suggesting that CSE may alleviate systemic inflammation. In conclusion, these findings indicate that CSE effectively reduces blood pressure and systemic inflammation in SHR without adversely affecting basal metabolism or organ function, with a stable and sustained antihypertensive effect lasting at least 4 weeks post-treatment, potentially involving a long-term mechanism, thus providing experimental evidence for CSE as a potential therapeutic candidate for hypertension.

**TABLE 2:** Comparison of pre- and post-intervention metabolic and physiological parameters between SHR and CSE-treated rats^*a*^

Index	SHR group_0w	SHR group_8w	CSE group_0w	CSE group_8w	*P*-value 1 (0w)	*P*-value 2 (8w)	*P*-value 3 (CSE 0w vs 8w)
Body weight (g)	320.5 ± 15.2	325.4 ± 16.1	318.7 ± 14.6	322.1 ± 15.3	0.82	0.45	0.38
Food intake (g/day)	21.50 ± 2.10	21.30 ± 1.95	21.80 ± 2.25	21.70 ± 2.00	0.62	0.72	0.68
Creatinine (μmol/L)	28.5 ± 3.1	35.2 ± 4.5	27.9 ± 2.8	30.1 ± 3.7	0.71	0.02*	0.11
BUN (mmol/L)	6.3 ± 0.6	7.7 ± 1.1	6.3 ± 0.7	6.7 ± 0.9	0.86	0.02*	0.27
CRP (mg/L)	4.3 ± 1.1	4.6 ± 1.3	4.5 ± 1.0	3.1 ± 0.8	0.68	0.01*	0.03*
LDL-C (mmol/L)	2.8 ± 0.5	2.9 ± 0.6	2.7 ± 0.4	2.6 ± 0.5	0.75	0.15	0.28
Blood glucose (mmol/L)	6.2 ± 0.7	6.5 ± 0.8	6.3 ± 0.6	6.1 ± 0.7	0.82	0.24	0.35
Serum sodium (mmol/L)	140.2 ± 3.5	142.1 ± 3.8	139.8 ± 3.2	138.4 ± 3.5	0.85	0.12	0.18
Serum potassium (mmol/L)	4.1 ± 0.3	4.0 ± 0.4	4.2 ± 0.3	4.1 ± 0.4	0.47	0.78	0.95
ALT (U/L)	44.7 ± 7.2	46.2 ± 8.1	43.9 ± 6.8	42.1 ± 7.3	0.78	0.34	0.41
AST (U/L)	81.2 ± 12.7	82.2 ± 13.6	80.1 ± 12.2	79.8 ± 12.9	0.62	0.45	0.84
Urine volume (mL/24h)	15.2 ± 3.1	15.8 ± 3.9	15.5 ± 3.3	16.2 ± 4.2	0.82	0.33	0.75
Water intake (mL/24h)	32.5 ± 4.2	33.1 ± 4.5	32.8 ± 3.9	33.6 ± 5.1	0.71	0.52	0.69

^*a*^ Data are presented as mean ± SD for normally distributed variables. *P*-value 1 compares SHR vs CSE atweek 0 (prior to intervention); *P*-value 2 compares SHR vs CSE after the 8-week intervention; *P*-value 3 compares the CSE group at pre- and post-intervention. All rats had free access to water and standard chow diet throughout the experiment. CRP, C-reactive protein; LDL-C, low-density lipoprotein cholesterol; ALT, alanine aminotransferase. Statistical significance was determined using Student’s *t*-test. *: *P* < 0.05.

### CSE significantly improves gut microbial diversity, composition, and metabolic function in SHR rats

**Fig 2:**
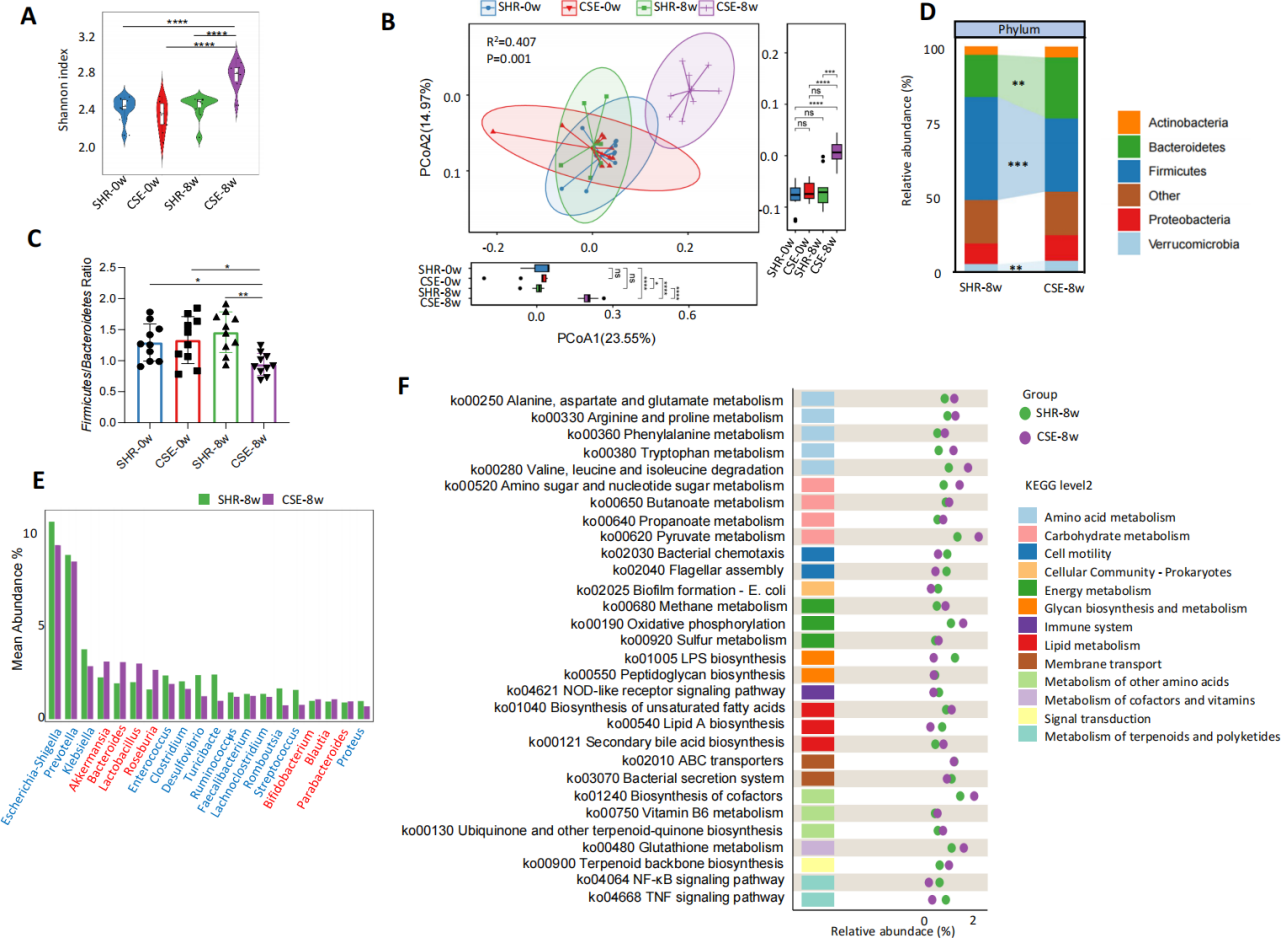
Effects of CSE on gut microbial diversity, composition, and function in SHR rats. (**A**) Comparison of α-diversity (Shannon index) of gut microbiota across groups. (**B**) PCoA based on weighted UniFrac distances shows overall differences in β-diversity. (**C**) Comparison of the *Firmicutes*/*Bacteroidetes* (F/B) ratio across groups. (**D**) Relative abundance and group-wise comparison of phylum-level microbial composition in the SHR-8w and CSE-8w groups. (**E**) Top 20 genera showing significant differences in relative abundance between SHR-8w and CSE-8w groups. (**F**) Functional prediction using 16S rRNA gene sequencing reveals significant differences in KEGG metabolic pathways between SHR-8w and CSE-8w groups, with the top 30 enriched pathways shown. SHR: untreated SHR rats; CSE: CSE-treated SHR rats. *n* = 10 per group. Data are presented as median with IQR for panels **A and B**, and as mean ± SD for panel **C**. Kruskal–Wallis test followed by Dunn’s multiple comparisons test was used for panels **A and B**; one-way ANOVA with LSD post-hoc test was used for panel **C**; two-tailed Wilcoxon rank-sum test with FDR correction was used for panels **D and E**. *: *P* < 0.05, **: *P* < 0.01, ***: *P* < 0.001, ****: *P* < 0.0001, ns: no significance.

16S rRNA sequencing revealed that at week 8, the Shannon index in the CSE group (CSE-8w) was significantly higher than that in the SHR control group (SHR-8w) and was also significantly increased compared with its own pre-intervention level, indicating marked improvement in microbial richness and evenness (Fig. 2A, *P* < 0.0001). PCoA based on weighted UniFrac distances revealed clear separation between CSE-8w and all other groups (PERMANOVA, *P* = 0.001; Fig. 2B), suggesting that CSE significantly reshaped the microbial community structure. At the phylum level, an 8-week intervention with CSE significantly reduced the *Firmicutes*/*Bacteroidetes* (F/B) ratio in SHR rats, indicating partial restoration of microbial homeostasis (Fig. 2C). The abundance of *Bacteroidetes* and *Verrucomicrobia* phyla was significantly increased in the CSE-8w group, whereas *Firmicutes* was reduced (Fig. 2D). Genus-level analysis further revealed an increased relative abundance of classic beneficial genera, such as *Akkermansia*, *Lactobacillus*, and *Roseburia*, along with reduced abundance of potentially pathogenic taxa, such as *Escherichia-Shigella* and *Klebsiella* in CSE-treated rats (Fig. 2E). Functional prediction analysis revealed a reconstructed metabolic potential of the gut microbiota following CSE intervention. In the CSE-8w group, metabolic pathways related to amino acid metabolism, carbohydrate metabolism, and energy metabolism were significantly upregulated, while inflammatory pathways, including TNF and NF-κB signaling and LPS biosynthesis, were downregulated (Fig. 2F). Collectively, these findings suggest that CSE modulates gut microbial diversity, composition, and metabolic function, potentially contributing to improved microbial homeostasis, reduced inflammation, and enhanced metabolic health in SHR rats.

### CSE alleviates systemic inflammation, reduces oxidative stress, and improves vascular function in SHR rats, potentially through modulation of the TLR4-eNOS-NO signaling pathway

**Fig 3:**
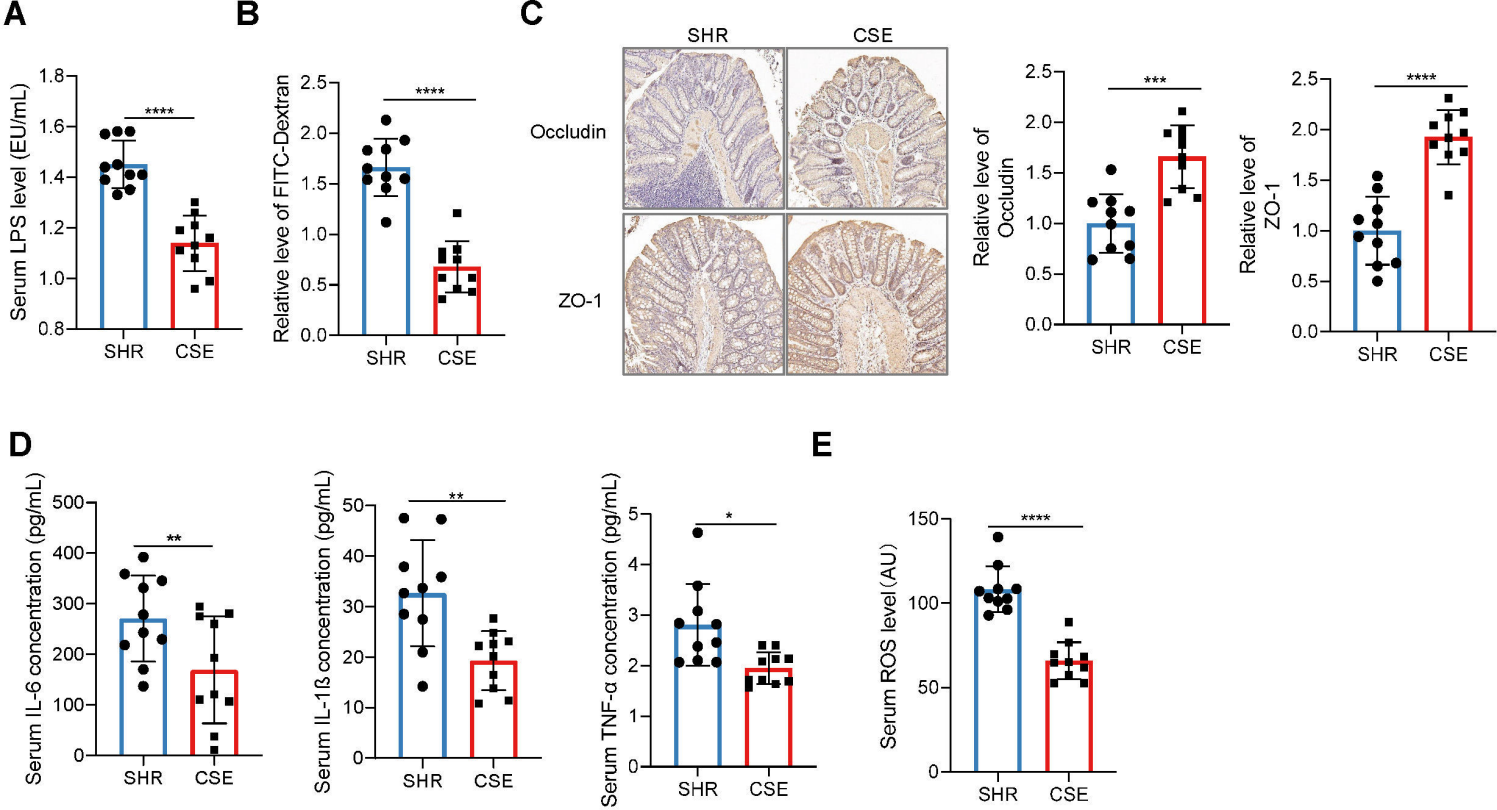
CSE alleviates systemic inflammation, improves intestinal barrier integrity, and reduces oxidative stress in SHR rats. (**A**) Comparison of serum LPS levels across groups. (**B**) Comparison of serum FITC-dextran fluorescence intensity among groups. (**C**) Representative immunohistochemistry (IHC) images and quantitative analysis of tight junction proteins ZO-1 and occludin in intestinal tissue across groups. (**D**) Comparison of serum levels of pro-inflammatory cytokines IL-6, IL-1β, and TNF-α across groups. (**E**) Comparison of serum ROS levels among groups. SHR: untreated SHR rats at week 8; CSE: CSE-treated SHR rats at week 8. *n* = 10 per group. Data are presented as mean ± SD for panels **A–E**. Student’s *t*-test was used for panels (**A–E**). *: *P* < 0.05, **: *P* < 0.01, ***: *P* < 0.001, ****: *P* < 0.0001.

**Fig 4:**
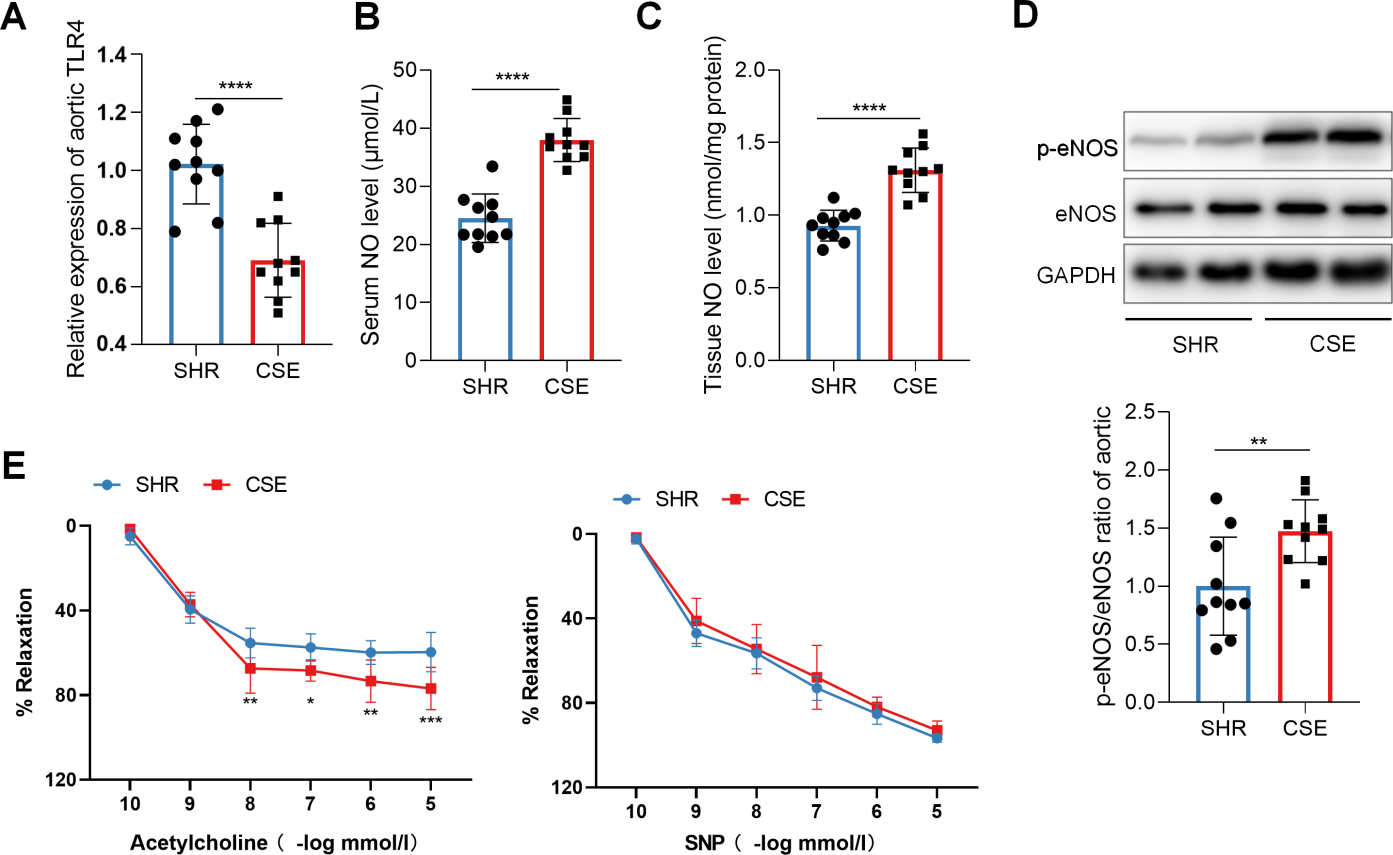
CSE downregulates TLR4 expression, enhances NO synthesis, and improves endothelium-dependent vasodilation in SHR rats. (**A**) Comparison of TLR4 mRNA expression in aortic tissues across groups. (**B**) Comparison of serum NO levels across groups. (**C**) Comparison of NO content in aortic tissues across groups. (**D**) Representative Western blot bands of p-eNOS and total eNOS, along with quantification of the p-eNOS/eNOS ratio in aortic tissues across groups. (**E**) Acetylcholine (or sodium nitroprusside)-induced vasodilation curves of aortic rings in each group. SHR: untreated SHR rats at week 8; CSE: CSE-treated SHR rats at week 8. *n* = 10 per group. Data are presented as mean ± SD for panels **A–E**. Student’s *t*-test was used for panels **A–D**; two-way ANOVA with LSD post-hoc test was used for panel **E**. *: *P* < 0.05, **: *P* < 0.01, ***: *P* < 0.001, ****: *P* < 0.0001.

Functional prediction of gut microbiota indicated that CSE may exert anti-inflammatory and antihypertensive effects via microbiota-mediated mechanisms. Given the critical role of intestinal barrier integrity in gut dysbiosis, systemic inflammation, and blood pressure regulation, we next assessed the effects of CSE on intestinal barrier function in SHR rats. The results showed that CSE treatment significantly improved intestinal barrier function in SHR rats, as evidenced by reduced serum LPS levels, decreased intestinal permeability, and increased expression of the tight junction proteins Occludin and ZO-1 in the colon (Fig. 3A through C). Concurrently, serum levels of inflammatory cytokines IL-6, IL-1β, and TNF-α were significantly decreased in the CSE group (Fig. 3D), along with a notable reduction in systemic reactive oxygen species (ROS) levels (Fig. 3E), indicating simultaneous attenuation of inflammation and oxidative stress. Previous studies have suggested that LPS can disrupt NO synthesis via inflammatory signaling, thereby contributing to elevated blood pressure. We therefore examined NO-related parameters in both serum and aortic tissue. First, mRNA expression of TLR4, the primary receptor for LPS, was significantly downregulated in the aorta of CS-8w rats (Fig. 4A). In parallel, CSE treatment significantly increased serum NO concentration, aortic NO content, and the p-eNOS/eNOS protein ratio (Fig. 4B through D). Notably, endothelium-dependent vasodilation in response to acetylcholine was significantly enhanced in the CSE-8w group (Fig. 4E), while vasodilation in response to sodium nitroprusside remained unaffected, suggesting that the improvement in vascular tone was due to enhanced NO bioavailability and restored endothelial function. Collectively, these findings demonstrate that CSE effectively alleviates systemic inflammation and oxidative stress while promoting NO production and endothelial-dependent vasorelaxation in SHR rats. These effects may represent a key mechanism by which CSE exerts its blood pressure-lowering action.

### Correlation and PERMANOVA analyses suggest that CSE regulates blood pressure primarily through the gut microbiota–inflammation–NO axis

**Fig 5:**
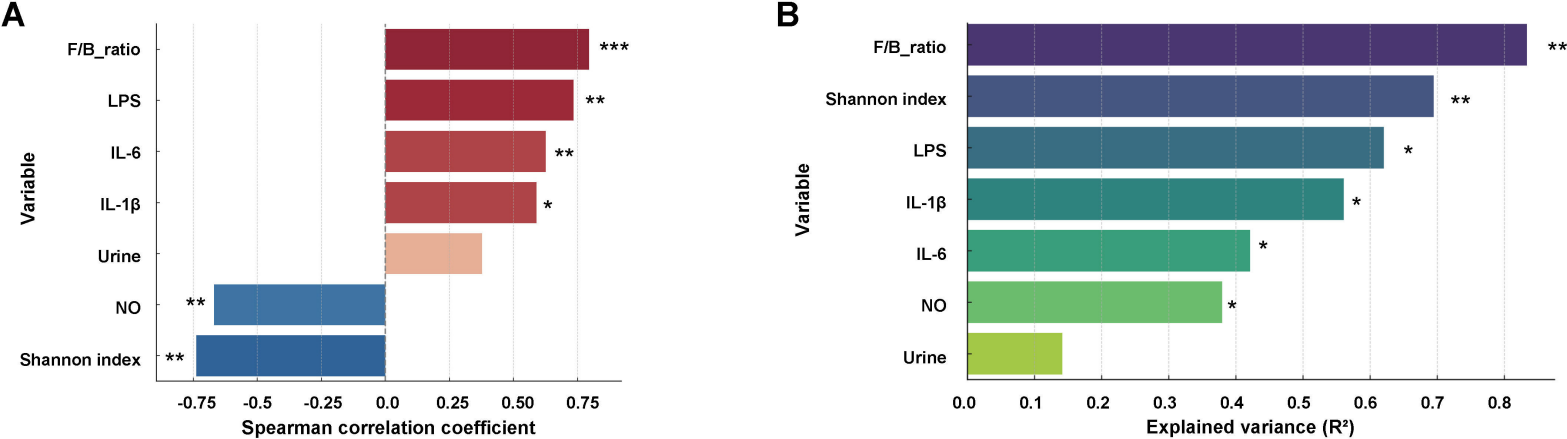
Causal analysis of the direct and indirect effects of CSE on blood pressure via the gut microbiota–inflammation axis and NO pathway. (**A**) Bar plot showing Spearman correlation coefficients (ρ) between blood pressure and various physiological variables. Red and blue bars indicate positive and negative correlations, respectively, with color intensity representing the strength of correlation. (**B**) PERMANOVA assessing the explanatory contribution of each variable to blood pressure variance. *P < 0.05, **P < 0.01, ***P < 0.001.

Given that corn silk has traditionally been associated with diuretic effects and that urine output is closely linked to blood pressure regulation, it was necessary to systematically evaluate the relative contributions of microbiota-related indices, inflammatory status, NO levels, and urine volume to blood pressure variation in SHR rats. To clarify the factors associated with blood pressure regulation in SHR rats, we first assessed correlations between microbial indices (Shannon diversity, F/B ratio), systemic inflammatory markers (LPS, IL-6, TNF-α), serum NO levels, urine output, and blood pressure (Fig. 5A). Blood pressure displayed significant negative correlations with microbiota diversity and NO levels, but positive correlations with the F/B ratio, LPS, and pro-inflammatory cytokines. No significant association was observed between blood pressure and urine output. Next, PERMANOVA was conducted to quantify the contribution of each factor to blood pressure variation (Fig. 5B). Microbial diversity, LPS, and inflammatory cytokines exhibited significant explanatory power, whereas urine output was not a contributing factor. These results support the conclusion that the antihypertensive effects of CSE are primarily mediated through modulation of gut microbiota composition, attenuation of endotoxemia and systemic inflammation, and enhancement of NO bioavailability, rather than through a diuretic mechanism traditionally attributed to corn silk.

### FMT validates the causal role of gut microbiota in the antihypertensive effects of CSE

**Fig 6:**
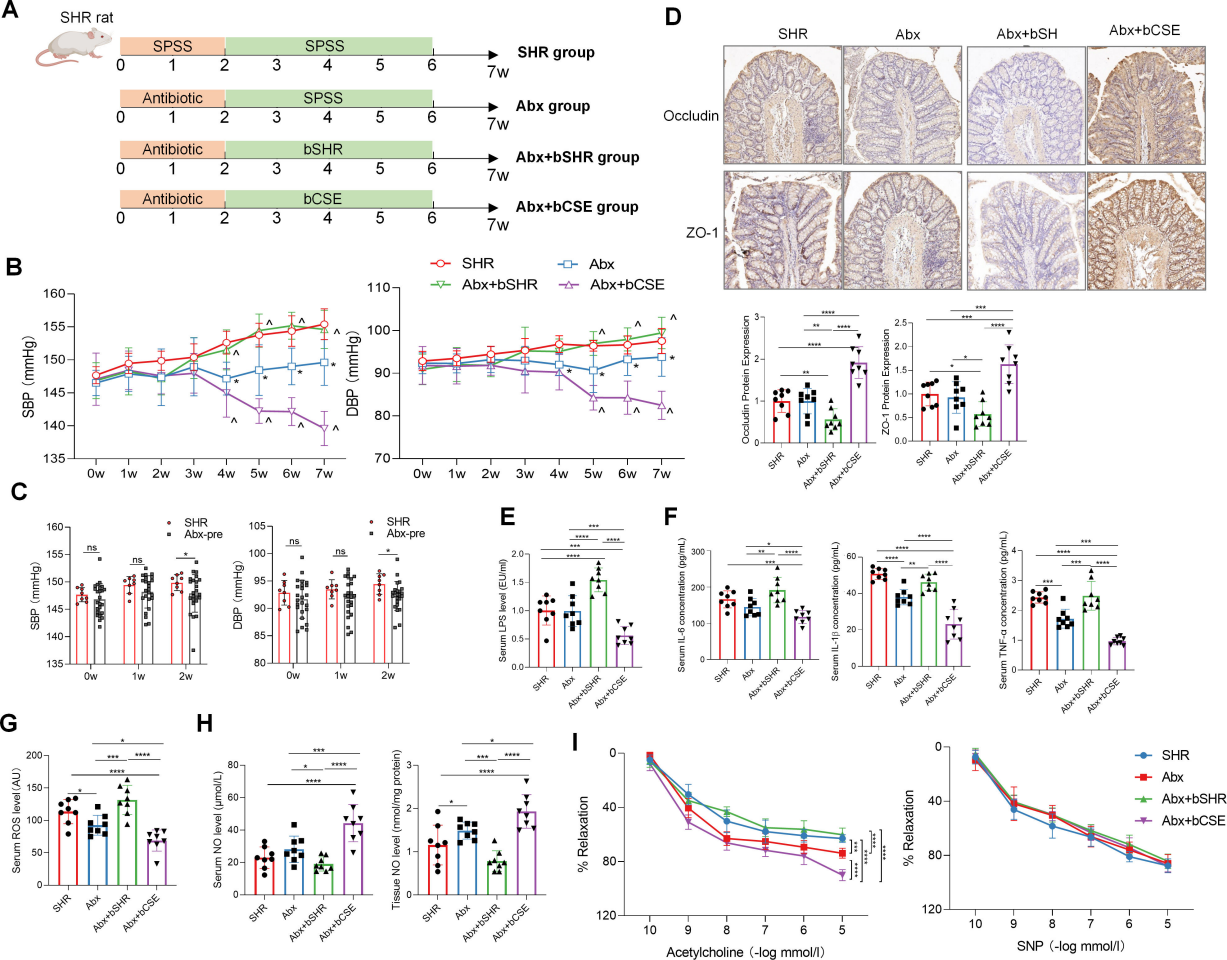
FMT validates the causal role of gut microbiota in mediating the effects of CSE on blood pressure and systemic inflammation. (**A**) Schematic of the FMT experimental protocol in SHR rats. (**B**) Dynamic changes in SBP and DBP among different groups during the experimental period. (**C**) Changes in SBP and DBP during the antibiotic pretreatment phase. Blood pressure was measured in untreated SHR rats (SHR group) and antibiotic-treated rats (Abx-pre group, including Abx, Abx+bSHR, and Abx+bCSE groups before FMT) at week 0 (prior to intervention), after 1 week, and after 2 weeks of antibiotic administration. (**D**) Representative IHC images and quantitative analysis of tight junction proteins ZO-1 and occludin in colonic tissue. (**E**) Serum LPS levels across groups. (**F**) Serum levels of pro-inflammatory cytokines IL-6, IL-1β, and TNF-αacross groups. (**G**) Serum ROS levels across groups. (**H**) NO levels in serum (left) and aortic tissue (right) across groups. (**I**) Endothelium-dependent (ACh-induced) and endothelium-independent (SNP-induced) relaxation curves of thoracic aortic rings in each group. SHR: untreated spontaneously hypertensive rats; Abx: rats with antibiotic-induced gut microbiota depletion; Abx+bSHR: Abx rats receiving fecal microbiota from SHR rats treated with PBS for 8 weeks; Abx+bCSE: Abx rats receiving fecal microbiota from SHR rats treated with CSE for 8 weeks. *n* = 8 per group. Data are presented as mean ± SD for panels B–I. Two-way ANOVA with LSD post-hoc test was used for panels **B and I**; one-way ANOVA with LSD post-hoc test was used for panels C–H. For B, *: *P* < 0.05, Abx vs. SHR, ^: *P* < 0.05, Abx+bSHR (bCSE) vs. Abx; For panels C–I, *: *P* < 0.05, **: *P* < 0.01, ***: *P* < 0.001, ****: *P* < 0.0001. ns: not significant.

To determine whether gut microbiota mediates the blood pressure-lowering effect of CSE, we established an antibiotic-treated SHR (Abx) model and conducted FMT using donor feces from SHR-8w (Abx+bSHR) and CSE-8w (Abx+bCSE) rats, thus clarifying the functional contribution of microbial alterations induced by CSE treatment (Fig. 6A). Results showed that antibiotic treatment did not induce any notable systemic adverse reactions or metabolic stress under the experimental conditions (Table 3). During the antibiotic pretreatment phase, depletion of gut microbiota by a broad-spectrum antibiotic cocktail led to a progressive reduction in blood pressure. Compared with untreated SHR rats, antibiotic-treated rats showed a decreasing trend in both SBP and DBP after 1 week of treatment, and this reduction became statistically significant after 14 days of antibiotic administration (Fig. 6B and C). These results indicate that gut microbiota depletion *per se* exerts a blood pressure-lowering effect in SHR. Upon FMT, rats receiving feces from CSE-treated donors exhibited further reductions in blood pressure, whereas those receiving feces from untreated SHR donors displayed increased blood pressure, comparable to that of the SHR group (Fig. 6B). Furthermore, FMT with microbiota from CSE-treated donors significantly improved intestinal barrier integrity, as evidenced by reduced serum LPS levels, increased colonic expression of the tight junction proteins occludin and ZO-1, and decreased expression of inflammatory cytokines, including IL-6, IL-1β, and TNF-α. In addition, serum ROS levels were significantly reduced. However, FMT with microbiota from SHR donors exacerbated these pathological indicators (Fig. 6D through G). In addition, microbiota from the CSE group significantly increased serum and aortic NO levels and improved endothelium-dependent relaxation of aortic rings in response to acetylcholine, whereas microbiota from SHR donors further impaired endothelial function. Notably, endothelium-independent relaxation induced by sodium nitroprusside was comparable across groups, indicating that these vascular effects were specifically attributable to enhanced endothelial NO bioavailability rather than changes in smooth muscle responsiveness. (Fig. 6H and I). Taken together, these results demonstrate that the antihypertensive effect of CSE in SHR is causally mediated by gut microbiota remodeling. Specifically, microbiota derived from CSE-8w rats contributes to blood pressure reduction by improving intestinal barrier function, alleviating systemic inflammation and oxidative stress, enhancing NO production, and restoring endothelial vasorelaxation capacity. In contrast, microbiota from untreated SHR rats exacerbates intestinal and vascular dysfunction, leading to increased blood pressure. These findings provide strong experimental evidence supporting the gut microbiota as a critical causal mediator in the antihypertensive effects of CSE and highlight its potential as a functional dietary intervention for hypertension management.

**TABLE 3:** Comparison of pre- (0w) and post-antibiotic treatment (2w) metabolic and physiological parameters

Index	Abx-pre group (0w)	Abx-pre group (2w）	*P*-value
Body weight (g)	318.3 ± 14.3	316.7 ± 15.1	0.91
Food intake (g/day)	21.3 ± 2.2	21.4 ± 2.1	0.99
Water intake (mL/24 h)	32.5 ± 4.8	32.2 ± 5.1	0.65

## DISCUSSION

In recent years, CSE has garnered increasing attention due to its richness in bioactive components, such as polysaccharides, polyphenols, and saponins. These compounds are considered to have potential prebiotic properties, and previous studies have reported their beneficial effects in blood pressure regulation, anti-inflammatory activity, and mild diuresis (22). However, whether the antihypertensive effects of CSE are mediated through modulation of the host gut microbiota remains poorly understood. In this study, we demonstrate for the first time that CSE acts as a potential prebiotic in SHR, significantly reducing blood pressure by enhancing microbial diversity, optimizing microbial community composition, improving intestinal barrier integrity, reducing serum LPS levels and systemic inflammation, and restoring NO-mediated vascular relaxation. Furthermore, integrative analyses of microbial, inflammatory, and vascular functional parameters revealed a coherent mechanistic framework in which improvements in gut microbiota structure were accompanied by reduced endotoxemia and systemic inflammation, enhanced NO bioavailability, and ultimately lower blood pressure. Consistently, FMT experiments demonstrated that microbiota from CSE-treated rats was sufficient to reproduce the antihypertensive phenotype, confirming that gut microbiota remodeling is an independent causal contributor to blood pressure regulation. Collectively, this study expands our understanding of the functional potential of CSE and provides the first mechanistic evidence that it exerts antihypertensive effects via gut microbiota modulation as a plant-derived prebiotic. These findings lay the theoretical foundation for the development of microbiota-targeted natural interventions in hypertension management.

A growing body of evidence has demonstrated that gut microbiota dysbiosis is commonly observed in both hypertensive patients and animal models, primarily characterized by decreased microbial diversity, an abnormally elevated *Firmicutes*/*Bacteroidetes* (F/B) ratio, and the proliferation of potential pathogenic bacteria (23). These microbial imbalances are thought to contribute to the onset and progression of hypertension by promoting systemic inflammation and endothelial dysfunction (24). Based on this understanding, we conducted a 4-week intervention with CSE followed by a 4-week post-treatment observation and systematically evaluated its effects on gut microbiota homeostasis and blood pressure regulation. The results showed that the treated rats exhibited significantly higher gut microbial diversity (Shannon index) and a markedly reduced F/B ratio compared with controls, indicating a general restoration of microbial homeostasis. Additionally, the relative abundances of beneficial genera, such as *Akkermansia* and *Lactobacillus* increased, while the abundance of potential pathogens like *Escherichia-Shigella* declined, further supporting the potential of CSE to promote healthy microbial reconstitution (19, 25). To further validate the independent antihypertensive effects mediated by gut microbiota remodeling, we specifically designed a combined strategy involving delayed sampling after treatment and FMT. Notably, even after discontinuation of the extract for 4 weeks, the treated rats maintained improved blood pressure levels and gut microbial structure, suggesting effects beyond the transient pharmacological action. More importantly, FMT from donor rats that had ceased CSE intervention for 4 weeks independently induced reductions in blood pressure, systemic inflammation, and restoration of NO signaling in recipient rats. These findings further confirmed that gut microbiota remodeling plays a causal and independent role in the antihypertensive effects of CSE, rather than being a secondary consequence of the treatment.

In addition, we paid particular attention to the diuretic mechanism frequently mentioned in traditional literature. Although some studies have suggested that CSE facilitates natriuresis and diuresis, thereby assisting in blood pressure reduction (26, 27), no significant change in urine volume was observed in the treated group before and after the intervention in our study. Further PERMANOVA analysis also indicated that urine output showed low explanatory power for changes in blood pressure and lacked statistical significance. This discrepancy may be attributed to differences in animal models (e.g., SHR vs renin-dependent hypertension models), dosing regimens, or variations in the proportion of active constituents and extraction methods. Given the multiple active components in CSE, its mechanism of action is likely multi-targeted and multi-pathway. Therefore, while our findings do not negate the possibility of a diuretic mechanism, they suggest that under the current model and intervention conditions, gut microbiota remodeling and downstream immune/metabolic modulation are likely the predominant pathways mediating the antihypertensive effect.

Hypertension is not only a hemodynamic disorder but is also commonly accompanied by a state of chronic low-grade systemic inflammation (28). Clinical and animal studies have shown that individuals with hypertension typically exhibit elevated serum levels of inflammatory markers, such as IL-6, TNF-α, and CRP, which are positively correlated with blood pressure levels (29). Impairment of the intestinal barrier allows bacterial components, such as LPS, to translocate into the bloodstream, activating the TLR4–NF-κB signaling pathway and inducing pro-inflammatory cytokine expression, thereby promoting persistent low-grade inflammation (30). Chronic inflammation, in turn, can suppress the activity of eNOS, reduce NO bioavailability, impair vasodilation, and increase peripheral vascular resistance. NO, as a key molecule maintaining vascular tone homeostasis, has been recognized as a central mediator in the development of hypertension, with its deficiency closely linked to elevated blood pressure (31). Increasing evidence suggests that gut microbiota dysbiosis can induce inflammation and impair NO signaling, thus acting as an important intermediary pathway for elevated blood pressure (32). Building upon this theoretical framework, our study provides further experimental evidence. Intervention with CSE significantly restored gut microbiota homeostasis and reduced serum LPS levels, accompanied by marked downregulation of inflammatory cytokines, such as IL-6 and TNF-α, suggesting that it may inhibit systemic inflammation by repairing the intestinal barrier. Further mechanistic analysis demonstrated that the treatment significantly increased serum NO levels, restored eNOS activity, and enhanced the p-eNOS/eNOS ratio, indicating improved endothelial function via enhanced NO pathway activity.

Corn silk, a traditional dual-purpose food and medicinal herb, has long been used as an adjuvant for lowering blood pressure, though its underlying mechanisms remain incompletely understood. In recent years, with the identification of its abundant bioactive components, such as polysaccharides, polyphenols, and flavonoids, its potential as a plant-derived prebiotic has attracted increasing attention, offering new perspectives for gut microbiota-based interventions in hypertension (19). In this study, a comprehensive analysis of microbiota composition suggested that CSE may function as a prebiotic to modulate blood pressure via gut microbial regulation. Among its active components, the non-starch polysaccharides found in corn silk (e.g., mannose, rhamnose, arabinose) structurally conform to the typical definition of prebiotics and can be metabolized by mucin-degrading commensals, such as *Akkermansia* (33). Additionally, its flavonoid and polyphenol constituents (e.g., apigenin, maysin) have bidirectional microbiota-modulating capacity, acting both as substrates for beneficial microbes and as selective antimicrobial agents to inhibit potential pathogens (34). These functional compounds may enrich the relative abundance of probiotic genera such as *Lactobacillus* and *Akkermansia* through nutritional selectivity and ecological optimization, thereby promoting the restoration of microbial homeostasis from dysbiosis (35).

Previous studies have also reported that corn silk treatment increases the abundance of *Akkermansia* and *Lactobacillus* while reducing *Escherichia*, a trend confirmed by our results (36). Our data demonstrated that CSE intervention significantly reshaped the gut microbiota in SHR rats, enriching probiotic genera such as *Akkermansia* and concurrently reducing potentially pathogenic taxa. These microbial signatures are consistent with the ecological markers typically observed in interventions using plant-derived prebiotics (25). Notably, the restructuring of the gut microbiota was accompanied by reductions in serum LPS, downregulation of pro-inflammatory cytokines, and restoration of NO signaling, suggesting that intestinal ecological repair may mediate blood pressure improvement through barrier restoration and systemic inflammation attenuation. Furthermore, the prebiotic effects of corn silk may partly depend on its capacity to serve as a substrate for the generation of SCFAs. Prior research has indicated that polysaccharides and polyphenols may promote SCFAs production, which can activate gut–immune–vascular signaling pathways and act as a key bridge in microbiota–host interactions (15). Therefore, we speculate that CSE lowers blood pressure by modulating community composition of commensal bacteria, enhancing intestinal barrier function, alleviating systemic inflammation, and ultimately activating the NO pathway. The specific role of microbial metabolites, such as SCFAs in this process, warrants further investigation using quantitative metabolomic and receptor-targeted validation approaches in future studies.

This study has several limitations that should be acknowledged. First, functional inferences based on PICRUSt2 were derived from 16S rRNA gene profiles rather than direct metagenomic or metabolomic measurements; therefore, the predicted pathways should be interpreted as exploratory. Second, although the antibiotic–FMT protocol allowed us to demonstrate a causal contribution of gut microbiota to the antihypertensive effects of CSE, broad-spectrum antibiotics themselves may transiently affect host physiology, and potential off-target effects cannot be completely excluded. Third, only male SHR rats were included, and possible sex-specific differences in gut microbiota composition and blood pressure responses cannot be ruled out. Finally, we did not directly quantify microbiota-derived metabolites, such as SCFAs or bile acids, which may represent important mediators linking CSE, gut microbiota, and vascular regulation. Future studies incorporating shotgun metagenomics, targeted metabolomics, and sex-balanced cohorts will be helpful to further refine and extend our mechanistic findings.

This study is the first to demonstrate that CSE can lower blood pressure by remodeling the gut microbiota—specifically, by enhancing microbial diversity, optimizing community composition, suppressing potential pathogens, and enriching the relative abundance of beneficial bacteria. These changes collectively contributed to the alleviation of systemic inflammation and the restoration of NO signaling, ultimately resulting in stable blood pressure reduction. This finding not only expands our understanding of the antihypertensive mechanism of corn silk but also provides strong support for its potential as a novel prebiotic candidate. As a plant extract with a long history of traditional use, wide availability, natural composition, and proven safety, CSE holds significant promise for development as a prebiotic with consistent gut-modulating and antihypertensive effects. It may be developed into dietary supplements, functional foods, or adjunct treatments for metabolic disorders—particularly for individuals seeking non-pharmacological options for blood pressure control. Furthermore, this study proposes and experimentally validates a mechanistic cascade in which gut microbiota modulation reduces inflammation, thereby restoring NO bioavailability and ultimately lowering blood pressure, and confirms the causal role of gut microbiota alterations through FMT experiments. This mechanistic insight not only sheds light on the functional basis of CSE but also provides a practical model for future investigations into the prebiotic potential of other plant-derived bioactives. Nonetheless, this study has certain limitations. The specific active components responsible for the observed effects remain to be identified, the long-term efficacy and generalizability in human populations require further evaluation, and the roles of microbial metabolites, such as SCFAs warrant deeper investigation. In summary, our findings offer direct experimental evidence that CSE may act as a prebiotic to modulate the gut microbiota and achieve blood pressure regulation, advancing the application of traditional plant-based compounds in microbiota-targeted interventions for chronic diseases, with significant theoretical and translational implications.

### Conclusion

This study provides the first experimental evidence that CSE exerts antihypertensive effects through modulation of the gut microbiota. In SHR, CSE intervention improved gut microbial diversity and enriched beneficial genera, such as *Akkermansia* and *Lactobacillus*, while reducing the relative abundance of potential pathogens. These microbial changes were associated with improved intestinal barrier function, reduced systemic inflammation, and enhanced NO-mediated endothelial function, ultimately contributing to blood pressure reduction. FMT experiments further verified the mediating role of gut microbiota in this process. Taken together, these findings identify CSE as a promising plant-derived prebiotic candidate for gut microbiota-targeted nutritional strategies in hypertension management. Future studies should focus on the identification of active compounds, long-term efficacy, and clinical applicability of CSE-based interventions.

## Data Availability

All data supporting the findings of this study are publicly available upon online publication, in accordance with ASM data-sharing policies. The data sets, including raw and processed sequencing files and all relevant metadata, have been deposited in the Dryad Digital Repository and can be accessed at the following DOI: https://doi.org/10.5061/dryad.34tmpg4w9.
